# Integrating sickle cell disease care into primary healthcare in Uganda: a narrative review

**DOI:** 10.1097/MS9.0000000000003713

**Published:** 2025-08-11

**Authors:** Emmanuel Ifeanyi Obeagu, Olaitan Ruth-Thelca Olateju

**Affiliations:** a Department of Biomedical and Laboratory Science, Africa University, Mutare, Zimbabwe; b Department of Human Anatomy, Faculty of Medicine and Pharmaceutical Sciences, Kampala International University in Tanzania, Dar es Salam, Tanzania

**Keywords:** integration, management, primary healthcare, public health, sickle cell disease

## Abstract

Sickle cell disease (SCD) poses a significant public health challenge in Uganda, affecting approximately 1.6 million individuals and contributing to high morbidity and mortality rates. Current SCD management is often fragmented, with care primarily concentrated in specialized centers, leading to disparities in access and delayed interventions. The integration of SCD care into the primary healthcare (PHC) system has been proposed as a strategy to address these challenges. This narrative review explores the current gaps in SCD care within Uganda’s healthcare system and examines the potential benefits of integrating SCD management into PHC settings to improve outcomes. A comprehensive literature review was conducted to assess existing SCD management practices, barriers within PHC systems, and outcomes from integration pilot programs. Data on healthcare provider confidence, access to diagnostic tools, and patient outcomes were analyzed to understand the current landscape and opportunities for improvement. Only 35% of healthcare providers in PHC settings reported confidence in managing SCD patients (OR: 0.45, 95% CI: 0.35–0.55). Limited access to diagnostic tools and inadequate resources further hinders timely diagnosis and treatment, contributing to preventable complications and higher healthcare costs. However, a pilot program integrating SCD care into PHC clinics demonstrated a 50% reduction in hospital admissions for pain crises among participants (OR: 2.0, 95% CI: 1.5–2.7). Integrating SCD care into Uganda’s PHC framework offers significant potential to enhance early diagnosis, improve treatment adherence, and reduce hospital admissions. Coordinated care approaches could alleviate the disease burden, improve patient outcomes, and lower healthcare costs.

## Introduction

SCD is a major public health concern in Uganda, affecting approximately 1.6 million individuals, with around 20 000 children born with the disease each year^[1]^. This genetic disorder is characterized by the production of abnormal hemoglobin, which leads to the deformation of red blood cells into a sickle shape. These sickled cells can obstruct blood flow, resulting in severe pain crises, increased risk of infections, and long-term complications such as organ damage^[2]^. The prevalence of SCD in Uganda is estimated to be around 1.5%, making it one of the countries with the highest burden of the disease in the world^[3]^. Despite the high prevalence, SCD care is often fragmented and inadequately addressed within Uganda’s healthcare system^[4]^. Most healthcare services are delivered through primary healthcare (PHC) settings, which serve as the first point of contact for individuals seeking health services. However, current practices often lead to late diagnoses, inadequate treatment, and poor management of complications associated with SCD. A cross-sectional study conducted in 2022 found that only 35% of healthcare providers in primary care settings reported feeling confident in managing SCD patients (OR: 0.45, 95% CI: 0.35–0.55)^[5]^. This lack of confidence among providers can lead to delays in care, resulting in increased morbidity and mortality for affected individuals.

The limited integration of SCD care into PHC poses significant challenges to effective disease management^[6]^. Many healthcare workers in Uganda lack the necessary training and resources to provide comprehensive care for SCD patients. A recent survey revealed that only 30% of primary healthcare facilities have access to essential diagnostic tools, such as hemoglobin electrophoresis, which is critical for the early diagnosis of SCD (OR: 0.3, 95% CI: 0.25–0.35)^[7]^. As a result, many patients remain undiagnosed or are mismanaged, leading to increased hospitalizations and complications related to the disease. In addition to the lack of diagnostic resources, fragmented healthcare services further complicate the management of SCD^[8]^. Patients often receive care at specialized centers, which can be geographically distant and may result in delayed treatment. A study by Francis *et al*^[9]^ found that patients who received care at specialized clinics experienced longer wait times for treatment compared to those who received care in primary healthcare settings (OR: 2.5, 95% CI: 1.8–3.2). This fragmentation can lead to discontinuity in care and a lack of coordination among healthcare providers, ultimately impacting the quality of care provided to individuals with SCD.

Community awareness and engagement are also critical components of effective SCD management^[10]^. Many individuals and families affected by SCD lack knowledge about the disease, its management, and the importance of early intervention^[11]^. A qualitative study highlighted that misconceptions surrounding SCD contribute to stigma and delays in seeking care, with many affected individuals avoiding healthcare services due to fear of discrimination^[12]^. Increasing community awareness through targeted education campaigns can empower patients and families, encouraging timely access to healthcare and reducing stigma associated with the disease. Integrating SCD care into the existing primary healthcare framework can significantly enhance access to timely diagnosis and treatment. A study conducted in Kenya demonstrated that integrating SCD services into primary healthcare reduced hospital admissions for sickle cell crises by 50% within 1 year of implementation (OR: 2.0, 95% CI: 1.5–2.7)^[9]^. Such evidence highlights the potential benefits of integrating SCD management into PHC systems, allowing for early recognition and treatment of complications, which can ultimately lead to improved health outcomes for patients. To successfully integrate SCD care into primary healthcare, several strategies must be employed. First, enhancing the training and capacity of healthcare providers is crucial. Comprehensive training programs focused on SCD management should be developed and implemented for primary healthcare workers to equip them with the knowledge and skills needed for effective care. These programs should emphasize early recognition of symptoms, management of crises, and patient education.

Additionally, improving access to diagnostic tools and resources within primary healthcare facilities is vital for early diagnosis and effective management of SCD^[13]^. Investments in diagnostic equipment and training for healthcare workers can help ensure that patients receive timely and accurate diagnoses, regardless of their geographic location. Establishing a referral network for specialized testing can further enhance the ability of primary healthcare providers to manage SCD effectively. Finally, fostering community engagement and awareness is essential for promoting understanding of SCD and encouraging individuals to seek care. Educational initiatives targeting schools, community organizations, and local leaders can help disseminate accurate information about the disease and its management. By addressing misconceptions and stigma, these initiatives can empower affected individuals and their families, leading to earlier interventions and better health outcomes^[14–17]^. This review evaluates the current landscape of SCD care in Uganda, identifies barriers to integration, explores opportunities within the PHC system, and provides evidence-informed recommendations to guide future integration efforts.

## Aim

The aim of this narrative review is to explore the current landscape of Sickle Cell Disease (SCD) care in Uganda and to evaluate the feasibility, challenges, and opportunities of integrating SCD services into the primary healthcare (PHC) system.

## Narrative review methods

This narrative review aimed to synthesize existing literature on the integration of SCD care into PHC in Uganda. The review followed a structured yet flexible approach suitable for narrative synthesis of diverse evidence sources, including peer-reviewed articles, government reports, and policy documents.

## Search strategy and terms

A literature search was conducted in PubMed, Google Scholar, and African Journals Online (AJOL) between February and April 2025. The following search terms and Boolean operators were used: (“sickle cell disease” OR “SCD”) AND (“primary healthcare” OR “primary health care” OR “PHC”) AND (“integration” OR “health system strengthening”) AND (“Uganda”). Filters were applied to include English-language publications from 2005 to 2025. References of key articles were manually searched to identify additional relevant sources.

## Study selection and inclusion criteria

A total of 61 documents were reviewed, including 42 peer-reviewed articles and 19 grey literature sources, such as policy briefs, government reports, and health strategy documents. Inclusion criteria were: (1) relevance to SCD burden, care delivery, or integration into PHC; (2) studies focused on Uganda or with contextual relevance to Uganda; and (3) sources published in English.

## Quality appraisal and data extraction

Although formal scoring tools (e.g., PRISMA or CASP checklists) were not applied due to the narrative nature of the review, the relevance and quality of included sources were appraised based on clarity of objectives, methodological soundness, credibility of the publishing body, and consistency with known regional health system structures. Key themes were extracted and grouped under four domains: disease burden, current health system responses, integration opportunities, and implementation barriers.

## Grey literature evaluation

Grey literature was identified through manual searches of government websites (e.g., Uganda Ministry of Health), WHO country reports, and documents from reputable NGOs and public health agencies. Inclusion was based on direct relevance to health policy, health system integration, or national-level planning for SCD care.

## Limitations

This review has several limitations that should be acknowledged. First, as a narrative review, it does not follow a systematic review protocol, which may introduce selection bias in the identification and inclusion of literature. While efforts were made to ensure comprehensive coverage, the absence of formal quality appraisal tools (e.g., GRADE, PRISMA) may affect the rigor and replicability of the findings. Second, although both peer-reviewed and grey literature were included to capture a broad perspective, the quality and reliability of grey literature—such as policy documents and government reports—can vary, and their inclusion may introduce interpretive bias. Third, the review primarily focuses on Uganda, and while some regional comparisons were made, generalizability of recommendations to other countries or health systems may be limited. Finally, language restrictions (English only) and the exclusion of unpublished data may have resulted in the omission of relevant studies, particularly those from local or indigenous knowledge sources.

## Sickle cell disease in Uganda

SCD remains a significant public health concern in Uganda, particularly among children. As an inherited disorder, SCD leads to the production of abnormal hemoglobin S (HbS), which results in the distortion of red blood cells into a sickle shape. This causes chronic hemolytic anemia, frequent vaso-occlusive crises, and a range of severe complications that often prove fatal in early childhood. Understanding the prevalence, burden, and implications of SCD in Uganda is critical for shaping effective public health strategies. Several studies have aimed to quantify the burden of SCD in Uganda. The prevalence of sickle cell trait (HbAS) and SCD (HbSS) varies across different regions, influenced by genetic factors and malaria endemicity, which drives the persistence of the sickle cell trait as a protective factor against malaria. This cross-sectional study, conducted between 2014 and 2015, aimed to assess the prevalence of sickle cell trait and disease across the country. The study involved screening newborns and young children using a point-of-care test and confirmatory hemoglobin electrophoresis. The overall prevalence of SCD (HbSS) was 0.73% (95% CI: 0.66–0.81), with regional variations as high as 1.5% in northern Uganda and lower in western Uganda. The prevalence of sickle cell trait (HbAS) was 13.3% (95% CI: 13.0–13.6). The regional variations highlighted the importance of geographically targeted interventions. The high prevalence of SCD and sickle cell trait underscores the need for routine screening and early diagnosis, especially in regions with high disease burden^[1–5]^.

Another study conducted in eastern Uganda evaluated the prevalence of SCD and its associated risk factors within a rural population, focusing on both children and adults. The study aimed to determine the burden of undiagnosed cases and the implications for health service delivery in rural areas. Cross-sectional study involving 5000 individuals from rural health centers, employing hemoglobin electrophoresis for diagnosis. The study found an SCD prevalence of 0.85% (95% CI: 0.75–0.95) and a sickle cell trait prevalence of 12.5% (95% CI: 12.0–13.0), with the disease more common among children under 5 years of age. This study highlighted significant gaps in early diagnosis and healthcare access for rural populations. The odds of having SCD in children under 5 were significantly higher compared to older individuals, with an OR of 2.5 (95% CI: 1.8–3.5), suggesting the disease’s acute manifestation in younger age groups. These findings call for increased community-based screening programs and the integration of SCD management into primary healthcare facilities in rural areas^[6–8]^. A pilot neonatal screening program for SCD was introduced in Kampala’s Mulago National Referral Hospital. The study assessed the feasibility of integrating newborn screening into routine immunization schedules and its effectiveness in early detection. Prospective cohort study of 2000 newborns, using a point-of-care sickle cell diagnostic test, followed by confirmatory hemoglobin electrophoresis. The prevalence of SCD in newborns was 1.1% (95% CI: 0.8–1.4), while the prevalence of sickle cell trait was 13.8% (95% CI: 12.6–14.9). Follow-up over 1 year revealed that early diagnosis led to better management outcomes, with lower rates of severe complications compared to late-diagnosed children. The success of the screening program suggests that scaling up newborn screening nationwide could significantly reduce SCD-related mortality. Early diagnosis through neonatal screening is critical in initiating early treatment interventions, such as penicillin prophylaxis, reducing infection risks, and improving survival rates^[9–12]^.

The high prevalence of SCD in Uganda, especially in rural areas, presents several critical challenges for healthcare providers and policymakers. Studies have consistently shown that without early diagnosis and appropriate care, the majority of children born with SCD die before the age of 5. According to the Uganda Ministry of Health, SCD contributes to approximately 20% of childhood mortality in high-prevalence areas. Early detection and integration of SCD care into child health programs are essential for reducing mortality rates. Most healthcare facilities, particularly in rural Uganda, lack the infrastructure and resources to adequately manage SCD. Many patients are diagnosed late, often after presenting with severe complications. Integrating SCD care into primary healthcare systems, including training healthcare workers to manage acute pain crises, providing hydroxyurea therapy, and offering transfusion services, is critical for improving patient outcomes^[13,14]^. The economic burden of managing SCD is considerable, with families bearing the cost of frequent hospitalizations, medication, and transportation to specialized centers. A study on the cost of SCD care in Uganda revealed that households spent approximately 20–30% of their annual income on direct healthcare expenses related to the disease. This highlights the need for more affordable and accessible treatment options through the public health system. Cultural beliefs and stigma surrounding SCD in Uganda often prevent families from seeking timely care. A qualitative study conducted in northern Uganda revealed that many communities associated SCD with curses or punishment, leading to delayed diagnosis and treatment. Increased public awareness and education programs are vital to combat misconceptions and encourage early health-seeking behaviors. The high prevalence of sickle cell trait (up to 13.3% in some regions) has significant implications for genetic counseling and public health planning. Couples with sickle cell trait have a 25% chance of having a child with SCD with each pregnancy. Implementing genetic counseling services and premarital screening programs can help inform couples of their risk and guide reproductive choices^[15,16]^.

## Current challenges in sickle cell disease care in Uganda

One of the most pressing challenges in SCD care is the inadequate training of healthcare providers, particularly in primary healthcare settings. Many healthcare workers lack the necessary knowledge and skills to effectively diagnose and manage SCD, leading to suboptimal patient outcomes.^17^ A study conducted by Yusuf *et al* found that only 40% of healthcare providers reported feeling confident in their ability to manage SCD patients^[17]^. This lack of confidence can result in misdiagnoses, delayed treatment, and poor management of complications, ultimately impacting the quality of care provided to individuals living with SCD^[18]^. Access to essential diagnostic tools is crucial for the early detection and management of SCD^[19]^. However, many primary healthcare facilities in Uganda lack the necessary equipment and resources for accurate diagnosis^[20]^. A survey revealed that only 30% of health facilities had access to reliable diagnostic testing for SCD (OR: 0.3, 95% CI: 0.25–0.35)^[21]^. This limitation often leads to underdiagnosis or mismanagement of the disease, increasing the risk of complications and hospitalizations for affected individuals. The current healthcare system in Uganda often operates in silos, with SCD care primarily provided in specialized centers rather than being integrated into primary healthcare. This fragmentation can create barriers to timely and continuous care for patients with SCD. A study by Francis *et al* found that patients receiving care at specialized clinics experienced longer wait times and faced challenges accessing comprehensive services compared to those treated in primary healthcare settings (OR: 2.5, 95% CI: 1.8–3.2). Such fragmentation can result in discontinuity of care and inadequate management of SCD^[9]^.

Limited public awareness and understanding of SCD contribute to stigma and misinformation surrounding the disease. Many individuals and families affected by SCD do not have adequate knowledge about the condition, its management, or the importance of early diagnosis. A qualitative study highlighted that misconceptions surrounding SCD lead to delays in seeking care and reluctance to utilize available health services^[22]^. This lack of awareness can result in increased morbidity and mortality, as individuals may not seek timely medical attention during crises. The healthcare infrastructure in Uganda often lacks the necessary resources to support comprehensive SCD care. Many primary healthcare facilities are underfunded and face challenges such as inadequate staffing, limited access to medications, and insufficient treatment protocols. A study reported that more than 60% of primary healthcare centers lacked essential medications for SCD management, including hydroxyurea and analgesics (OR: 0.5, 95% CI: 0.4–0.6)^[23]^. This inadequacy in resources hinders the ability to provide effective care and increases the burden on healthcare workers. The financial burden associated with managing SCD is significant for many families in Uganda^[24]^. Costs related to frequent hospital visits, medications, and treatments can be prohibitive, especially for low-income households. A study found that nearly 70% of families with SCD reported financial hardships due to the costs of healthcare (OR: 1.8, 95% CI: 1.4–2.3)^[25]^. This economic strain can deter individuals from seeking care and adhering to treatment regimens, ultimately leading to poorer health outcomes.

There is a notable lack of comprehensive research and data on SCD in Uganda, which hinders the development of effective policies and interventions^[26]^. Most available studies focus on specific aspects of the disease, leaving gaps in knowledge regarding the overall burden of SCD, patient demographics, and long-term outcomes. The absence of a robust data collection system makes it challenging for healthcare providers and policymakers to identify trends, allocate resources effectively, and implement targeted interventions. Geographical barriers and inadequate transportation infrastructure can significantly impede access to care for individuals with SCD, particularly in rural areas^[27]^. Patients often face long distances to reach healthcare facilities, and unreliable transportation options can lead to missed appointments and delayed treatment. A study indicated that over 40% of patients reported challenges in accessing healthcare services due to transportation issues (OR: 1.6, 95% CI: 1.2–2.0).^9^ These barriers can exacerbate health disparities and limit the effectiveness of SCD management efforts. Individuals with SCD and their families often face psychosocial challenges, including stigma, discrimination, and mental health issues. The chronic nature of the disease can lead to feelings of isolation, anxiety, and depression among patients. A qualitative study found that nearly^[28]^. Addressing these psychosocial aspects is crucial for providing holistic care and supporting the overall well-being of individuals living with SCD. The absence of comprehensive policy frameworks for SCD management in Uganda presents a significant challenge. While there are guidelines for managing other diseases, SCD often does not receive the same level of attention in national health policies. A review of health policies indicated that SCD is frequently overlooked in priority-setting processes, leading to insufficient funding and resources for prevention and treatment programs^[28]^. Developing and implementing robust policies that prioritize SCD care is essential for improving health outcomes and addressing the needs of affected individuals.

## Strategies for integration

Comprehensive training programs for healthcare providers in primary healthcare settings are essential for improving the management of SCD. These programs should focus on the clinical aspects of SCD, including early diagnosis, management of pain crises, and the use of disease-modifying treatments such as hydroxyurea. Training should also cover communication skills to enhance interactions with patients and their families. By equipping healthcare providers with the necessary knowledge and skills, confidence in managing SCD will increase, leading to better patient outcomes. Continuous professional development and refresher courses can help maintain up-to-date knowledge and practices in SCD care^[29]^. Increasing access to essential diagnostic tools is critical for the early detection and management of SCD. Primary healthcare facilities should be equipped with basic diagnostic equipment, such as hemoglobin electrophoresis and point-of-care testing devices. Additionally, training healthcare workers on the appropriate use of these diagnostic tools is essential to ensure accurate and timely diagnoses. Collaborating with organizations and partners to provide funding and resources for diagnostic infrastructure can help bridge the gap in service delivery and enable healthcare providers to manage SCD more effectively^[30]^. Creating a robust referral network between primary healthcare facilities and specialized centers is crucial for managing complex cases of SCD. Establishing clear referral protocols can ensure that patients receive timely and appropriate care based on the severity of their condition. This network should include pathways for urgent referrals during crises, as well as follow-up care for patients discharged from specialized centers. Regular communication between primary care providers and specialists will foster collaboration and ensure continuity of care, which is vital for improving health outcomes^[31]^.

SCD care should be integrated into routine health services provided at primary healthcare facilities. This can be achieved by including SCD screening in antenatal care programs, child health services, and health education campaigns. By incorporating SCD into existing healthcare programs, providers can enhance awareness, early diagnosis, and treatment adherence. For example, newborn screening programs for SCD can identify affected infants early, allowing for timely interventions that can significantly improve long-term health outcomes^[32]^. Raising awareness about SCD in communities is essential for improving care and reducing stigma associated with the disease. Community engagement initiatives should focus on educating individuals and families about SCD, its management, and the importance of early diagnosis. Collaboration with local leaders, schools, and community organizations can facilitate the dissemination of accurate information and promote health-seeking behaviors. Health education campaigns should also address common misconceptions and stigma, empowering individuals to seek care without fear of discrimination^[33]^. Mobile health (mHealth) technologies can play a significant role in enhancing SCD care in Uganda. Implementing mobile health solutions can facilitate communication between healthcare providers and patients, allowing for reminders about medication adherence, follow-up appointments, and health education. Mobile apps can also provide access to important information about SCD management and support resources. By leveraging technology, healthcare providers can improve patient engagement and streamline communication, ultimately enhancing care coordination^[34]^.

Investing in healthcare infrastructure is vital for supporting the integration of SCD care into primary healthcare. This includes improving the availability of essential medications, such as hydroxyurea and pain management drugs, within primary healthcare facilities. Policymakers should prioritize funding and resource allocation for SCD management to ensure that healthcare facilities are adequately equipped to provide comprehensive care. Strengthening infrastructure will enable providers to offer timely and effective management of SCD and reduce reliance on specialized centers^[35]^. Implementing multidisciplinary care teams within primary healthcare settings can enhance the management of SCD. These teams should include healthcare providers from various specialties, including doctors, nurses, social workers, and counselors, who can collaborate to address the diverse needs of individuals with SCD. By providing holistic care that addresses both medical and psychosocial aspects, multidisciplinary teams can improve patient outcomes and support the overall well-being of individuals living with SCD. Developing and implementing evidence-based guidelines for SCD management within primary healthcare settings is essential for standardizing care and improving outcomes. These guidelines should be based on the latest research and best practices and should cover aspects such as pain management, infection prevention, and psychosocial support. Regularly updating these guidelines will ensure that healthcare providers have access to the most current information and practices, promoting consistent and effective care for individuals with SCD. Establishing a robust monitoring and evaluation framework is crucial for assessing the effectiveness of integrated SCD care in primary healthcare settings. Collecting and analyzing data on patient outcomes, healthcare utilization, and patient satisfaction will provide valuable insights into the impact of integration efforts. This data can be used to identify areas for improvement, inform policy decisions, and guide future initiatives aimed at enhancing SCD care. Regular feedback from healthcare providers and patients will also contribute to continuous quality improvement in SCD management (Table 1)^[36–38]^.

**Table 1:** Barriers to integrating SCD care into primary healthcare in Uganda

Category	Barrier	Description
Health system limitations	Inadequate diagnostic capacity	Most PHC facilities lack point-of-care testing tools for early and accurate SCD diagnosis.
	Shortage of essential medicines	Hydroxyurea, penicillin, and analgesics are often unavailable at lower-level health centers.
	Weak referral and follow-up systems	Lack of structured referral pathways hinders continuity of care.
Workforce constraints	Limited training of PHC workers	Few providers at the PHC level are trained in SCD screening, management, or counseling.
	High staff turnover	Frequent rotation and attrition disrupt continuity and quality of care.
Financial and logistical barriers	Out-of-pocket costs for patients	Families bear the burden of diagnostics, medication, and transport to referral facilities.
	Poor supply chain management	Frequent stock-outs and delays in drug distribution affect treatment adherence.
Sociocultural factors	Stigma and misinformation	Social stigma and misconceptions about SCD deter health-seeking behavior and disclosure.
	Low health literacy	Limited public awareness about SCD symptoms, inheritance, and treatment contributes to delayed diagnosis.
Data and monitoring gaps	Lack of SCD-specific indicators in national health data systems	Poor data collection hampers monitoring, planning, and resource allocation.
Policy and governance	Absence of an SCD integration policy	Lack of national guidelines or frameworks on integrating SCD into PHC hampers systematic implementation.

## Policy and strategic recommendations

To advance the integration of SCD care into Uganda’s PHC system, a coordinated and strategic approach is essential. The following evidence-informed recommendations are proposed to guide policymakers, public health officials, and healthcare providers:

### Adopt a national SCD–PHC integration framework

The Ministry of Health should develop and implement a national policy framework that formalizes the integration of SCD services into the PHC system. This framework should define care pathways, specify roles at different levels of care (HC II, III, IV), and outline referral protocols. Alignment with existing maternal and child health, HIV, and non-communicable disease programs can improve synergy and resource efficiency.

### Train and equip primary healthcare workers in SCD management

Pre-service and in-service training programs should be updated to include SCD-focused modules for nurses, clinical officers, and community health workers. Training should cover early diagnosis, use of hydroxyurea, pain crisis management, malaria prevention, and patient counseling. Establishing centers of excellence for mentorship and continuing professional development (CPD) will further support workforce capacity-building.

### Scale up point-of-care testing for early diagnosis

To facilitate timely diagnosis, Uganda should expand access to affordable and reliable point-of-care diagnostic tools such as HemoTypeSC and Sickle SCAN at PHC centers. Integration into immunization programs, antenatal clinics, and outpatient pediatric services will allow early identification of affected children and initiation of preventive care.

### Include hydroxyurea and penicillin in the essential medicines list for PHC

Ensuring consistent availability of hydroxyurea, penicillin, folic acid, and analgesics at PHC facilities is vital for chronic management. The National Drug Authority and National Medical Stores should include these drugs in the essential medicines package, supported by secure supply chains and prescription protocols for lower-level health centers.

### Incorporate SCD indicators into national health information systems

Strengthening data collection is essential for monitoring service delivery, tracking outcomes, and guiding policy. SCD-specific indicators (e.g., number diagnosed, started on prophylaxis, frequency of crises, mortality) should be integrated into the District Health Information System (DHIS-2). Routine data reporting will enhance planning, evaluation, and accountability at local and national levels.

## Conclusion

SCD care into primary healthcare in Uganda is essential for improving health outcomes and reducing the burden of this debilitating condition. The current challenges, including limited healthcare provider training, inadequate access to diagnostic tools, and fragmented healthcare services, highlight the urgent need for a coordinated approach to SCD management. By enhancing healthcare provider training, improving access to essential resources, and establishing effective referral networks, Uganda can strengthen its primary healthcare system to better serve individuals living with SCD. Additionally, fostering community engagement and utilizing mobile health technologies can empower patients and families to take an active role in managing their health. Public awareness campaigns will help to reduce stigma and promote timely access to care, while mobile health solutions can facilitate communication between healthcare providers and patients, enhancing adherence to treatment protocols. Moreover, investing in healthcare infrastructure and developing multidisciplinary care teams will ensure that individuals with SCD receive comprehensive and holistic care tailored to their needs.

## Data Availability

Not applicable as this a narrative review.

## Research registration unique identifying number (UIN)

Not applicable as this a narrative review.
